# The Efficacy of Stellate Ganglion Blockade in Herpetic Neuralgia: A Case Report

**DOI:** 10.7759/cureus.43805

**Published:** 2023-08-20

**Authors:** Thalis A Asimakopoulos, Athanasia A Gika, Panayotis S Kekelos, Irene C Kouroukli

**Affiliations:** 1 1st Department of Anesthesiology and Pain Medicine, National and Kapodistrian University of Athens School of Medicine, Athens, GRC; 2 Anaesthesiology Department and Pain Clinic, Hippocratio General Hospital of Athens, Athens, GRC

**Keywords:** stellate ganglion block (sgb), stellate ganglion blockade, vzv, case report, hyperalgesia, blockade, postherpetic neuralgia, stellate ganglion, herpes zoster

## Abstract

The varicella-zoster virus can reactivate in patients with impaired cell-mediated immunity, resulting in herpes zoster (HZ) and sometimes in herpetic neuralgia (HN). Stellate ganglion blockade (SGB) has been studied for the treatment of HN and postherpetic neuralgia (PHN), but its effectiveness in preventing PHN in patients with acute HZ is not well-established. Here, we present a case of a 75-year-old woman who underwent SGB for HN of the right upper limb. The patient had been treated with antivirals and various analgesics, but adverse effects due to analgesics led to discontinuation of them. After the first application of SGB, the patient experienced significant pain reduction, and after a second application, complete remission of pain was achieved. Nine months later, the patient remained symptom-free and without PHN. The therapeutic potential of SGB in the treatment of HN and its role in preventing PHN requires further investigation.

## Introduction

Varicella zoster virus (VZV) reactivation results in the infectious condition known as herpes zoster (HZ). Following the resolution of the initial VZV infection (chicken pox), the virus typically lays dormant in the sensory ganglia of the cranial and spinal nerves, but it can be reactivated in patients with impaired cell-mediated immunity due to advanced age or immunosuppressive medical conditions and malignancies [1].

Herpetic neuralgia is a painful condition caused by this reactivation. It is a common complication of HZ and can be difficult to manage with conventional analgesics.

It is known that the sympathetic nervous system plays a significant role in the pathogenesis of pain. Anatomic and chemical couplings between sympathetic postganglionic and afferent neurons may develop after nerve damage or tissue inflammation as a result of collateral sprouting in the peripheral and dorsal root ganglia and the upregulation of functional adrenoceptors [2]. Given that individuals with postherpetic neuralgia (PHN) experience worsened allodynia and higher pain following local administration of adrenergic agonists, some evidence point to a relationship between sympathetic activity and pain in PHN as well. Hence, administering sympathetic nerve blocks might stop the interactions between the sympathetic nervous system and the senses, relieving pain [3].

Stellate ganglion blockade (SGB) has been studied as a treatment for PHN, but its effectiveness in preventing PHN in patients with acute HZ is not well-established. Here, we present a case of a 75-year-old woman who underwent SGB for herpetic neuralgia of the right upper limb.

## Case presentation

**Figure 1:**
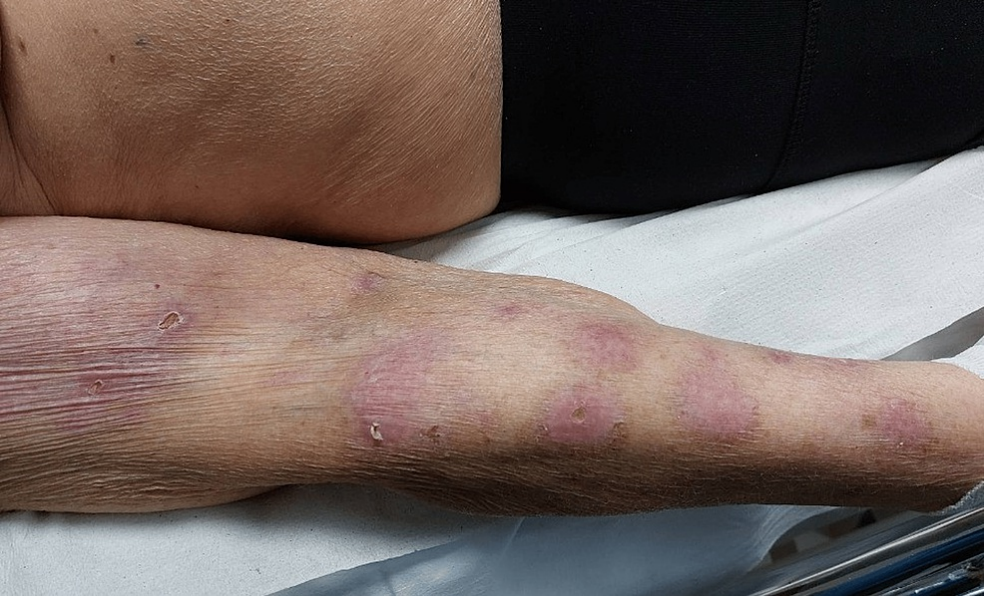
Rash of the upper limb

The patient presented to our Pain Clinic suffering from herpetic neuralgia of the right upper limb at the distribution of C5-C6-C7 dermatomes, for one month after shingles rash (Figure 1). From her medical history, she had lymphoma for which she had received chemotherapy. She had been receiving antiviral therapy for one week with the onset of the rash. The patient reported causalgia, numbness, and needle sensation in the area of distribution of the rash. She was initially treated with gabapentin 300mg twice a day and paracetamol 1g twice a day without significant relief. The patient reported pain according to a numerical rating scale (NRS) 7 out of 10. Decrease of pain occurred one hour after taking analgesics. DN4 questionnaire recording gave 6 out of 10 confirming the presence of neuropathic pain. The dose of gabapentin was increased to 300mg three times a day, and the combination of paracetamol 325mg+tramadol 37.5mg was added gradually up to three times a day regimen. However, the patient could not tolerate this new therapy due to adverse effects (dizziness, nausea, vomiting) and both regimens were discontinued.

**Figure 2:**
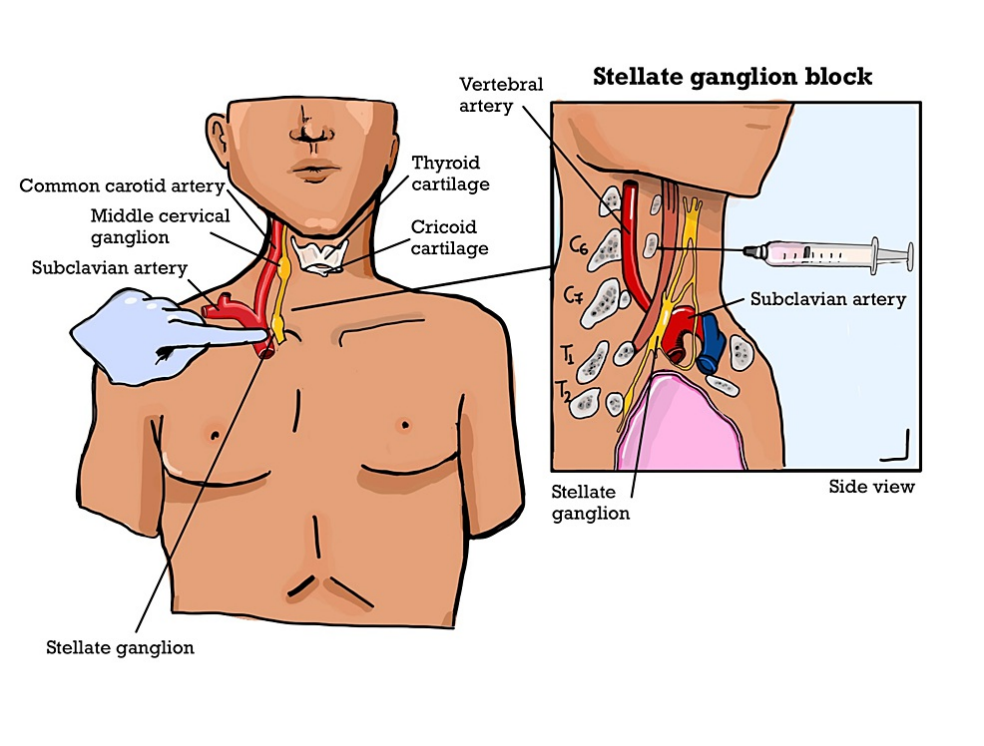
Anatomy of the stellate ganglion and the stellate ganglion block landmark technique This image was designed and created by the authors.

It was decided to perform SGB with the traditional approach based on anatomical landmarks according to the literature by identifying the sixth cervical vertebral tubercle (Chassaignac’s tubercle). This can be easily succeeded by locating the cricoid cartilage and moving the fingers until they contact this palpable tubercle (Figure 2). The injectate spreads from the stellate ganglion along the longus colli. By directly visualizing vascular structures and the target structure, ultrasound-guided SGB increases procedure safety [4,5], but regrettably it was not offered in our facility. The procedure was performed under sterile conditions with the patient in a supine position setting a pillow under her arms. An intravenous (IV) line was established. The skin was cleaned with an antiseptic solution at the injection site. A 23-gauge needle was then inserted into the target area using a luer lock syringe and the solution of 7ml of local anesthetic 0.5% ropivacaine with adrenaline in concentration 1:200.000 (to avoid intravascular penetration) and dexamethasone 8mg was injected slowly while monitoring the patient for any adverse reactions. For the monitoring we used ECG, pulse oximeter and non-invasive blood pressure (NIBP).

**Figure 3:**
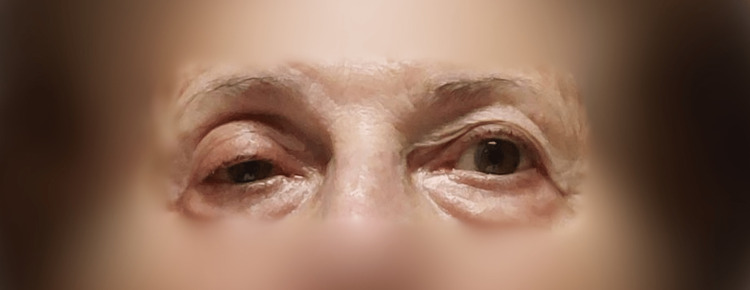
Horner’s syndrome

Patient established Horner’s syndrome immediately (miosis, ptosis, anhidrosis, enophthalmos) (Figure 3). The patient reported minimal discomfort during the procedure and slight hoarseness which lasted approximately 45 minutes. After the first session, the patient showed significant pain reduction (NRS 3) while completely discontinuing analgesics. 48 hours after the first procedure a second one was applied using local anesthetic only (7ml of 0.5% ropivacaine with adrenaline 1:200.000), and the patient showed complete remission of pain (NRS 0) reporting mild numbness. The patient is still being followed up by our clinic and already nine months later she is symptom-free and not taking analgesics, without having any symptoms of PHN as well.

## Discussion

Blocking the sympathetic nerves in the head, neck, and upper extremity is a key way that SGB works to enhance blood flow to these areas. The treatment of sympathetically mediated pain in the head, neck, and upper extremity is also successful with SGB. The inhibition of neuronal connections in the sphere of innervation of SGB can be used to explain its analgesic effects [6]. 

Many studies suggest that nerve blocks, specifically SGB, can be effective in preventing PHN in patients with acute herpes zoster. A systematic review and meta-analysis by Kim et al. found that nerve blocks significantly reduced the incidence of PHN compared to control groups [1]. Chen et al. also conducted a systematic review and meta-analysis of interventions for preventing postherpetic neuralgia in patients with herpes zoster. They found that antiviral therapy can significantly reduce the incidence of postherpetic neuralgia and improve patient outcomes [7].

In addition, Makharita et al. demonstrated that early SGB can reduce the incidence of PHN in patients with acute herpes zoster and facial pain [8]. Finally, a mini-review by Jeon et al. further highlights the therapeutic potential of SGB in orofacial pain, which can include pain associated with herpes zoster [6].

According to Aggarwal et al., for PHN of the face, neck, and upper limb distribution, SGBs with local anesthetics or its pulsed radiofrequency ablation (RFA) have been utilized successfully; nevertheless, their effectiveness is greater and longer-lasting if the PHN lasts less than a year [9].

Postherpetic neuralgia can be safely and effectively treated using ultrasound-guided SGB and shock wave therapy, which can considerably reduce pain levels in patients with the condition. Ding et al. also suggest that treatment of facial and upper limb PHN with SG pulsed radiofrequency is secure and efficient. That is a procedure worth advocating for [5].

Kim et al. published that PHN incidence is not reduced by SGB, however numerous blocks may have positive effects. Somatic blocks, including paravertebral and repeated/continuous epidural blocks, prevent and lessen the likelihood of PHN. Early nerve block may have stronger preventative effects when administered repeatedly or continuously than when given only once [1]. Future research will need consensus-based definitions of PHN, clinical success cutoffs that specify treatment outcomes, and standardized outcome assessment instruments, including measures of physical and psychological functioning.

Early management of infection and pain is likely to reduce the risk of postherpetic neuralgia. Treatment for this syndrome of chronic pain is challenging. The initial options are tricyclic antidepressants (amitriptyline) and antiepileptic medications (pregabalin, gabapentin). Interventional therapies, such as corticosteroid and local anesthetic injections into the epidural space, have an impact on the immediate pain but have minimal efficacy in avoiding postherpetic neuralgia. A sympathetic ganglion blockade may be attempted when conservative treatment for postherpetic neuralgia is unsuccessful in giving adequate relief. Spinal cord stimulation may be an option if severe pain persists [10].

For selecting the single best interventional treatment, the available evidence is insufficient. Subcutaneous injection of botulinum toxin A or triamcinolone, transcutaneous electrical nerve stimulation, peripheral nerve stimulation, and SGB are advised initially, followed by paravertebral block and pulsed radiofrequency, considering invasiveness, cost, and safety [11].

The oldest and most popular type of sympathetic block used today is SGB. Its applications range from PHN, intractable angina, complex regional pain syndrome (CRPS) [12,13] hyperhidrosis, arrhythmias, and hot flashes to post traumatic stress disorder (PTSD) [14-17]. There are promising case report studies for the use of SGB in the management of acute postoperative pain after amputation and phantom limb pain, although the scientific community has to undertake more in-depth research on these applications [18].

Based on these findings, nerve blocks, and specifically SGB, can be a useful intervention in preventing PHN in patients with acute herpes zoster affecting the head and upper limbs. Future research can further explore the optimal timing and frequency of nerve block interventions to maximize their efficacy in preventing PHN.

## Conclusions

To conclude, previous studies have shown that SGB can reduce the incidence of PHN in patients with HZ, although the quality of evidence is rated as low to moderate. The effectiveness of SGB in preventing PHN in patients with acute HZ is still not well-established, and more high-quality studies are needed to confirm its effectiveness. Nonetheless, our case report demonstrates the use of SGB in relieving pain in a patient with herpetic neuralgia of the right upper limb. The patient had previously tried multiple conventional analgesics, but none provided significant relief. The SGB procedure resulted in complete remission of pain and a sustained improvement in symptoms up to nine months later. This case report supports the use of SGB as a therapeutic option for managing herpetic neuralgia as well as the prevention of PHN in patients who do not respond to conventional analgesics. Healthcare providers should discuss the potential risks and benefits of SGB with their patients before considering this procedure.

## References

[1] Kim HJ, Ahn HS, Lee JY (2017). Effects of applying nerve blocks to prevent postherpetic neuralgia in patients with acute herpes zoster: a systematic review and meta-analysis. Korean J Pain.

[2] Wu CL, Marsh A, Dworkin RH (2000). The role of sympathetic nerve blocks in herpes zoster and postherpetic neuralgia. Pain.

[3] Choi B, Rowbotham MC (1997). Effect of adrenergic receptor activation on post-herpetic neuralgia pain and sensory disturbances. Pain.

[4] Wang C, Yuan F, Cai L, Lu H, Chen G, Zhou J (2022). Ultrasound-guided stellate ganglion block combined with extracorporeal shock wave therapy on postherpetic neuralgia. J Healthc Eng.

[5] Ding Y, Yao P, Li H, Han Z, Wang S, Hong T, Zhao G (2019). CT-guided stellate ganglion pulsed radiofrequency stimulation for facial and upper limb postherpetic neuralgia. Front Neurosci.

[6] Jeon Y (2016). Therapeutic potential of stellate ganglion block in orofacial pain: a mini review. J Dent Anesth Pain Med.

[7] Chen N, Li Q, Yang J, Zhou M, Zhou D, He L (2014). Antiviral treatment for preventing postherpetic neuralgia. Cochrane Database Syst Rev.

[8] Makharita MY, Amr YM, El-Bayoumy Y (2012). Effect of early stellate ganglion blockade for facial pain from acute herpes zoster and incidence of postherpetic neuralgia. Pain Physician.

[9] Aggarwal A, Suresh V, Gupta B, Sonthalia S (2020). Post-herpetic neuralgia: a systematic review of current interventional pain management strategies. J Cutan Aesthet Surg.

[10] van Wijck AJ, Wallace M, Mekhail N, van Kleef M (2011). Evidence-based interventional pain medicine according to clinical diagnoses. 17. Herpes zoster and post-herpetic neuralgia. Pain Pract.

[11] Lin CS, Lin YC, Lao HC, Chen CC (2019). Interventional treatments for postherpetic neuralgia: a systematic review. Pain Physician.

[12] Wie C, Gupta R, Maloney J, Pew S, Freeman J, Strand N (2021). Interventional modalities to treat complex regional pain syndrome. Curr Pain Headache Rep.

[13] O’Connell NE, Wand BM, Gibson W, Carr DB, Birklein F, Stanton TR (2016). Local anaesthetic sympathetic blockade for complex regional pain syndrome. Cochrane Database Syst Rev.

[14] Lipov E, Ritchie EC (2015). A review of the use of stellate ganglion block in the treatment of PTSD. Curr Psychiatry Rep.

[15] Hayase J, Patel J, Narayan SM, Krummen DE (2013). Percutaneous stellate ganglion block suppressing VT and VF in a patient refractory to VT ablation. J Cardiovasc Electrophysiol.

[16] Guttuso T Jr (2013). Stellate ganglion block for treating hot flashes: a viable treatment option or sham procedure?. Maturitas.

[17] Gunduz OH, Kenis-Coskun O (2017). Ganglion blocks as a treatment of pain: current perspectives. J Pain Res.

[18] Annabi EH, Arefieg J, Shiller S (2017). Stellate ganglion blockade. Treatment of Chronic Pain Conditions: A Comprehensive Handbook.

